# Split-Cre Complementation Indicates Coincident Activity of Different Genes *In Vivo*

**DOI:** 10.1371/journal.pone.0004286

**Published:** 2009-01-27

**Authors:** Johannes Hirrlinger, Anja Scheller, Petra G. Hirrlinger, Beate Kellert, Wannan Tang, Michael C. Wehr, Sandra Goebbels, Andreas Reichenbach, Rolf Sprengel, Moritz J. Rossner, Frank Kirchhoff

**Affiliations:** 1 Interdisciplinary Centre for Clinical Research (IZKF), N05 Neural Plasticity, Faculty of Medicine, University of Leipzig, Leipzig, Germany; 2 Department of Neurogenetics, Max Planck Institute of Experimental Medicine, Göttingen, Germany; 3 DFG Research Center for Molecular Physiology of the Brain, Göttingen, Germany; 4 Section of Biology, Chemistry and Pharmacy, Free University of Berlin, Berlin, Germany; 5 Paul Flechsig Institute for Brain Research, Faculty of Medicine, University of Leipzig, Leipzig, Germany; 6 Department of Molecular Neurobiology, Max Planck Institute for Medical Research, Heidelberg, Germany; Institute of Infectious Disease and Molecular Medicine, South Africa

## Abstract

Cre/LoxP recombination is the gold standard for conditional gene regulation in mice *in vivo*. However, promoters driving the expression of Cre recombinase are often active in a wide range of cell types and therefore unsuited to target more specific subsets of cells. To overcome this limitation, we designed inactive “split-Cre” fragments that regain Cre activity when overlapping co-expression is controlled by two different promoters. Using transgenic mice and virus-mediated expression of split-Cre, we show that efficient reporter gene activation is achieved *in vivo*. In the brain of transgenic mice, we genetically defined a subgroup of glial progenitor cells in which the *Plp1-* and the *Gfap*-promoter are simultaneously active, giving rise to both astrocytes and NG2-positive glia. Similarly, a subset of interneurons was labelled after viral transfection using *Gad67*- and *Cck*1 promoters to express split-Cre. Thus, split-Cre mediated genomic recombination constitutes a powerful spatial and temporal coincidence detector for *in vivo* targeting.

## Introduction

The central nervous system (CNS) as the most complex organ in mammals is composed of a large variety of heterogeneous cell populations. To characterize distinct cell types, neuroscientists take advantage of the fact that many neuronal and glial subsets can be defined by the expression of a unique combination of genes. Broadly expressed cell markers, such as the neuronal nuclei antigen NeuN, the glial fibrillary acidic protein (GFAP), myelin proteolipid protein (PLP) and the fractalkine receptor CX3CR1 (which identify neurons, astrocytes, oligodendrocytes and microglia, respectively), are commonly used in immunohistochemical analysis of brain development or disease. Moreover, a plethora of transgenic mice has been generated, in which the promoters of these and other “cell type-specific” genes drive the expression of fluorescent proteins or Cre recombinase in the nervous system.

The Cre/LoxP system [1] has been extensively used for cell fate mapping [2]–[5] and numerous studies exist in which Cre/loxP was used to analyse the cell-type specific function of a gene by conditional gene ablation or induction [6], [7]. This includes analysis of mechanisms of spatial memory in CA1 neurons of the hippocampus or the function of peroxisomes in oligodendrocytes [8], [9].

Despite this impressive success of cell-specific gene targeting and recent progress in the temporal control of Cre activity [10], the expression pattern of a single promoter activity is often insufficient to genetically define a distinct neural cell type [11]. To overcome this limitation of Cre/LoxP-mediated DNA-recombination, we added a second dimension of recombination control by designing a novel “split-Cre” system based on the complementation of Cre protein fragments. For this purpose, we constructed fusion proteins of a constitutive protein-protein interaction domain with either N- or C-terminal Cre fragments (designated NCre and CCre, respectively). We selected the GCN4-coiled coil domain as the interaction domain since it has been shown to form stable dimers [12] and to enable functional complementation of dihydrofolate reductase *in vitro* and in E. coli [13] as well as of tobacco etch virus protease *in vitro* [14]. Each of these split-Cre proteins alone was unable to catalyse DNA recombination. However, they readily assembled into a functional enzyme when co-expressed in the same cell *in vivo* in transgenic mice, or by viral infection. Using a Cre-reporter mouse strain we demonstrate that Cre activity persistently labels cells defined by transient, but coincident expression of NCre and CCre during embryogenesis. Split-Cre is thus a versatile tool to study transient cell populations during development and disease processes and to precisely genetically target distinct cell populations in the CNS as shown here, but additionally in other organs.

## Results

### Generation of split-Cre-proteins

**Figure 1:**
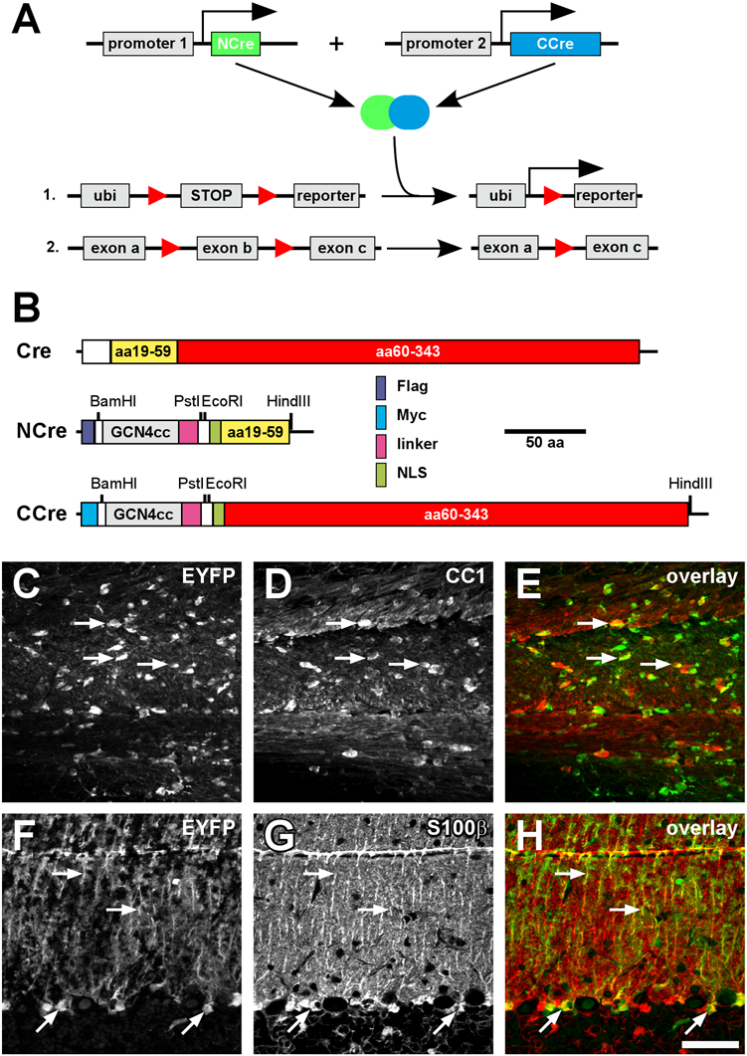
A: Principle of split-Cre complementation. NCre- and CCre-coding sequences are placed under the control of two different promoters (promoter 1 and 2, respectively). Only if both promoters are active, functional complementation takes place and LoxP (red triangles) – flanked sequences are recombined, thereby activating reporter genes (A1) or excising exons for knock-out strategies (A2). Ubi: ubiquitously expressed promoter (in our study the ROSA26 promoter). B: Design of fusion proteins. NCre- and CCre-proteins were constructed by fusing amino acids 19–59 (NCre) or amino acids 60–343 (CCre) of the sequence of Cre recombinase to the constitutive active coiled-coil interaction domain of the yeast transcription factor GCN4 (GCN4cc). Immunotags (Flag, Myc), a linker sequence as well as a nuclear localization sequence (NLS) were also added. C–H: Functional complementation of Cre activity in transgenic mice. C–E: NCre and CCre were transgenically expressed in mice using the PLP promoter. Transgenic mouse lines expressing NCre (line PCND) and CCre (line PCCR) were crossed to ROSY reporter mice and analysed for recombination with in the corpus callosum (C). Slices were counterstained with the oligodendroglial marker CC1 (D). The overlay is shown in E. Arrows point to examples of CC1-positive oligodendrocytes, which show also expression of the EYFP reporter. F–H: NCre and CCre were transgenically expressed in mice using the GFAP-promoter. Transgenic mouse lines expressing NCre (line GCNV) and CCre (line GCCF) were crossed to ROSY reporter mice and analysed for recombination in the cerebellum (F). Bergmann glia cells were stained with antibodies against S100β (G). The overlay is shown in H. Arrows highlight S100β-positive cells which show recombination. The scale bar in H corresponds to 50 µm and applies to panels C–H.

To establish a Cre-based complementation system (“split-Cre”), which allows recombination of LoxP-flanked DNA sequences depending on the simultaneous activity of two promoters (Fig. 1A), the coding sequence of Cre recombinase was cut into two complementation-competent fragments [15], [16] coding for amino acids residues 19–59 and 60–343. Both Cre fragments were fused to the constitutively dimerizing coiled-coil leucine zipper domain of the yeast transcriptional activator GCN4 [12] to force the association of split-Cre fragments, thereby enhancing Cre activity by functional complementation. In addition, immunotags, a semi-flexible linker and a nuclear localization sequence were added to both Cre fragments. The resulting split-Cre fusion proteins were named NCre and CCre, respectively (Fig. 1A, B). Functional complementation of these constructs was tested first in COS, CHO and PC12 cells. High recombination efficiency was observed whenever both NCre and CCre proteins were co-expressed, while no recombination was detected when either NCre or CCre were expressed alone (Fig. S1).

### Functional complementation of split-Cre in vivo

To analyse whether split-Cre complementation induces recombination activity *in vivo*, we generated transgenic mice expressing NCre and CCre under the control of the mouse proteolipid protein (PLP) and the human glial fibrillary acidic protein (GFAP) promoter (see Fig. S2A for transgene constructs). These glial promoters were selected since previous fate-mapping studies of glial lineages demonstrated PLP- as well as GFAP-promoter activity in progenitor cells during fetal development of the brain. It is, however, not clear whether this occurs in the same cells or in different population of progenitor cells. We obtained three lines of PLP-NCre-mice (PCNA, PCNC, PCND), two lines of PLP-CCre mice (PCCK, PCCR), four lines of GFAP-NCre- (GCNQ, GCNT, GCNV, GCNW) as well as one GFAP-CCre-mouse line (GCCF) (Text S1). Successful complementation and functional recombination was detected in triple-transgenic mice (using the reporter line ROSA26-LoxP-Stop-LoxP-EYFP [17]; ROSY) when (1) both NCre and CCre were targeted to oligodendrocytes by the PLP promoter (Fig. 1C–E; Fig. S3) or when (2) both fragments were expressed in astrocytes driven by the GFAP promoter (Fig. 1F–H; Fig. S5). For comparative evaluation of split-Cre vs. full-length Cre induced recombination in transgenic mice, founder-dependent line-to-line variability of expression patterns inherent to mouse transgenesis has to be considered. This variation affects split-Cre more than normal Cre-mice due to the need of two transgenes. Taken these idiosyncrasies into account, split-Cre revealed a similar pattern and extend of reporter gene expression (Fig. S3, S4 and S5; and compare to [2]–[4], [18]). The slightly reduced number of recombined cells in PLP-NCre x PLP-CCre mice compared to the maximal recombination achievable in PLP-Cre mice (Fig. S3, S4) can be attributed to not completely overlapping expression patterns of the PLP-NCre and PLP-CCre transgenes in the respective mouse lines. Therefore, we conclude that the NCre/CCre complex catalyzes DNA-recombination in cells co-expressing both proteins.

### Distinct populations of glial progenitors identified by Cre complementation

**Figure 2:**
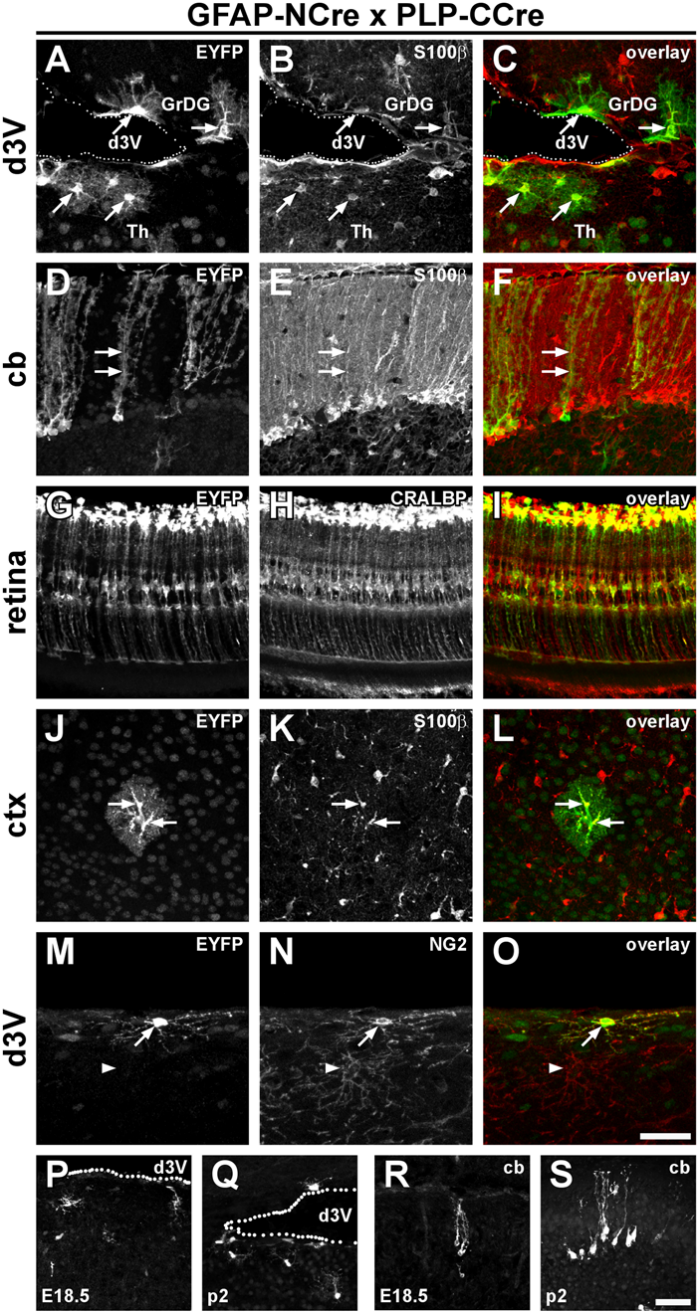
A combination of PLP- and GFAP-promoter drives recombination mainly in astrocytes and NG2-cells. Transgenic mouse lines expressing NCre under the GFAP promoter and CCre under the control of the PLP promoter were crossed to ROSY-reporter mice and analysed for recombination in different brain regions (A, D, G, J, M). Slices were counterstained using the astroglial marker S100β (B, E, K), the Müller cell marker cellular retinaldehyd binding protein (CRALBP; H) as well as NG2 (N). Overlays are shown in C, F, I, L and O. Most cells showing recombination in these mice stain also for the astroglial marker (examples are marked by arrows in A–L). A subpopulation of EYFP-labeled cells is positive for NG2 (arrow in M–O), but not all NG2 cells express the reporter EYFP (arrow head in M–O). P–S: Developmental appearance of labeled cells in the juxtaventricular zone close to the dorsal 3^rd^ ventricle (P, Q) and in the cerebellum (R, S). Shown are examples from embryonic day 18.5 (P, R) and postnatal day 2 (Q, S). The dotted lines in P, Q show the approximate border of the ventricle. Mouse lines GCNV x PCCK (G–I); GCCF x PCND (M–O), GCNT x PCCK (P–S) and GCNT x PCCR (all others) were used for this analysis. The scale bars in O and S correspond to 50 µm and apply to panels A–O and P–S, respectively. d3V: dorsal third ventricle; GrDG: granular layer of the dentate gyrus; Th: thalamus; cb: cerebellum; ctx: cortex.

Previous fate-mapping studies have shown that Cre-dependent recombination driven by GFAP or PLP promoters during embryonic development labels distinct glial and neuronal cell populations [2]–[4], [18]. Here, we asked whether both promoters are active in separate lineages or whether coincident activity can highlight a distinct neural cell type or cellular subpopulation. For that purpose, we analysed the Cre-complementation of the GFAP-NCre and PLP-CCre transgenes in ROSY reporter mice (Fig. 2). We could identify Cre-induced EYFP expression in various brain regions. Co-immunostaining for the reporter EYFP and the astroglia marker protein S100β together with distinct morphology revealed that most of the cells in which recombination had taken place were *bona fide* astrocytes (Fig. 2). Interestingly, the recombination pattern suggested that cerebellar Bergmann glial cells as well as a glial population at the juxtaventricular zone were derived from progenitors with simultaneous GFAP- and PLP-gene activity (Fig. 2A–F). We counted the fraction of EYFP-labeled Bergmann glial cells as a percentage of all S100β-positive cells in the Purkinje cell layer of the cerebellum and found 25±8% labeled cells (mean±sd; n = 4 mice; 400 cells counted in total). In contrast, cortex (Fig. 2J–L), hippocampus, striatum or cerebellar white matter (Fig. S6) showed only a few labeled cells. Interestingly, another radial glial cell type, the Müller cells of the retina, exhibited a similarly high recombination frequency as the Bergmann glia (Fig. 2G–I). Although labeled cells were mainly identified as astrocytes, we also detected throughout the brain of all transgenic crossings cells, which were identified as NG2 glia due to their expression of the chondroitin sulphate proteoglycan NG2 [19]–[21] (Fig. 2M–O).

To identify the developmental stage when the PLP- and GFAP promoters are active in the same cell, we stained brain tissue for EYFP-expression at different time points during development. While no reporter expression could be detected at E12.5 and E15.5 (data not shown), EYFP positive cells could be detected at embryonic day 18.5 (E18.5) in the juxtaventricular zone close to the dorsal 3^rd^ ventricle (Fig. 2P), the cerebellum (Fig. 2R) and in the midbrain (data not shown). By postnatal day 2 (P2), the cells in the cerebellum and in the midbrain acquired the typical morphology of Bergmann glial cells and astrocytes, respectively (Fig. 2S).

In conclusion, these experiments demonstrate that split-Cre reports coincident gene activities *in vivo*. Functional complementation of N- and C-terminal Cre fragments is achieved even when co-expression of both peptides is only transient due to the control by distinct promoter elements.

### Interneurons expressing GAD67 and CCK can be targeted by split-Cre in vivo using adeno-associated viruses

**Figure 3:**
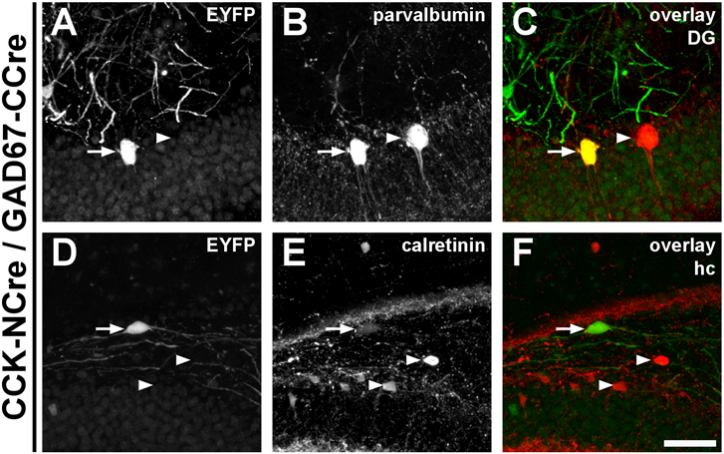
Functional complementation of split-Cre *in vivo* after viral infection. Adeno-associated viral vectors (AAV) containing NCre or CCre under the control of the CCK- and GAD67-promoter, respectively, were injected into brains of ROSY reporter mice and recombination was analysed by EYFP expression. A population of interneurons showing DNA recombination was identified (A, D) that express either parvalbumin (B, arrow) or calretinin (E, arrow). Arrowheads mark cells which are positive for the respective marker protein, but show no recombination. The scale bar in F corresponds to 50 µm and applies to all panels. DG: dentate gyrus; hc: hippocampus.

Due to its flexibility viral infection has become a common tool to genetically label or modify cells *in vivo*. In contrast to transgenic mice, viruses can be generated fast and can also be applied at defined time points of development or disease progression. Therefore, we decided to test whether the split-Cre fragments would be able to functionally recombine when introduced by adeno-associated viral (AAV) vectors. In this set of experiments we focussed on interneurons, since the identification of interneuron subpopulations is difficult [22], [23]. As interneuronal promoter elements, we selected those for the GABA-synthesizing enzyme glutamate acid decarboxylase (GAD67) and for the neurohormone cholecystokinin (CCK; see Fig. S2B for viral constructs). For control purposes, we first tested whether mixtures of viruses expressing NCre and CCre from the same promoter would indeed label the respective cell type. For comparison, we also used mixtures of GFAP-NCre and GFAP-CCre encoding AAV viruses and detected specific recombination in many GFAP-positive astrocytes of the injected region (Fig. S7A–F). Similarly, co-injection of GAD67-NCre and GAD67-CCre viruses into the hippocampus resulted in the labelling of interneurons of which a subpopulation could be immunostained with the marker parvalbumin (Fig. S7G–L). Finally, co-injection of CCK-NCre and GAD67-CCre viruses into the hippocampus resulted in recombination in interneurons which could be labelled by parvalbumin (Fig. 3A–C) as well as by calretinin (Fig. 3D–F). No recombination could be observed when NCre- or CCre expressing viruses were injected alone, irrespective of the regulatory element used to drive the expression (data not shown). These data thus show that adeno-associated viral vectors can be successfully used to transfer split-Cre fragments. The split-Cre system is thus a powerful tool for inducing DNA-recombination *in vivo* and targeting cell populations defined by the simultaneous expression of two genes.

## Discussion

### Split-Cre: A powerful new tool for mouse genetics

Our novel split-Cre complementation system introduces a second dimension to the cell-type specific control of DNA-recombination *in vivo.* We divided the Cre recombinase into two fragments and fused both to strong protein interaction domains. The resulting fusion proteins (termed NCre and CCre) were able to complement each other thus reconstituting functional Cre recombinase in neurons and glia of a living mouse. Transgenic co-expression of NCre and CCre and subsequent functional complementation induced a pattern of reporter gene expression as observed with intact Cre recombinase driven by the same promoters. In transgenic as well as in virus-infected animals, distinct cell populations defined by simultaneous activity of two different genes were targeted. The coincident detection of gene activities combined with efficient, permanent DNA-modification makes our approach a useful extension of existing DNA recombinase based methods. In combination with the plethora of existing mouse lines with LoxP-flanked genes, split-Cre provides a unique platform, which will allow more sophisticated genetic experiments to analyze cell lineage or cell heterogeneity in the future.

### Technical considerations

Functional complementation of split proteins is a powerful approach and has already been widely used for the analysis e.g. of protein-protein-interactions. The functional, bimolecular complementation of diverse proteins such as the green fluorescent protein (GFP), firefly luciferase, dihydrofolate reductase, β-galactosidase or tobacco etch virus protease has become an important assay system *in vitro* [24]. The reconstitution of fluorescent protein fragments has become particularly important since the formation of the functional protein complex can be directly visualized in living cells [25], [26]. However, fluorescent protein complementation still constitutes only a transient signal [27]. In contrast, stable and long-lasting labeling and modification of cells by DNA-recombination can be achieved using fragment complementation based on the Cre/LoxP system.

For forced dimerization of the Cre fragments, we selected a yeast interaction domain to decrease the risk of interaction with endogenous murine coiled-coil proteins. For example, GCN4 coiled-coil domains do not interact with c-myc of the host [28]. Although we cannot exclude the possibility that our constructs bind to endogenous proteins and interfere with cellular physiology, we did not observe any abnormalities in the transgenic mice generated, with respect to cage behaviour, breeding performance and expression of marker proteins. Therefore, split-Cre complementation by itself does not appear to alter mouse physiology. Previously, Cre fragments have been fused to FKBP12 (FK506-binding protein) and FRB (binding domain of the FKBP12-rapamycin-associated protein) for dimerization [15], [16]. However, to induce binding here, rapamycin must be used which is disadvantageous for two reasons: (1) it is an immunosuppressant and can interfere with animal physiology; (2) its use for analysis of the CNS is limited due to its poor ability to traverse the blood brain barrier. Indeed, no recombination was observed within the brain [16].

The detection and extend of functional Cre complementation is determined by several parameters which have to be considered carefully for interpretation of data:

1. Temporal relations within the split-Cre system. While the cell-specific expression levels of NCre and CCre is primarily determined by the activities of the chosen promoters, the visualization of complementation depends on the kinetics of complementation, DNA recombination, reporter gene transcription and translation as well as the time needed to accumulate enough reporter protein to allow detection. Furthermore, after cessation of short-lived promoter activities remaining split-Cre mRNA and protein could evoke DNA recombination even in the absence of promoter activity. From our *in vitro* data and our experiences with recombination induction and reporter detection in the tamoxifen-inducible CreERT2/loxP system [29], we estimated a temporal resolution of about 1 to 2 days for the split-Cre system.
2. Specificity of transgenic or viral expression. It is a common observation that mouse lines generated with the same transgene construct can differ in their expression and show a mosaic pattern. This is mainly attributed to the site and copy number of integration of the transgene within the genome. This mouse line-to-mouse line variability is potentiated in the split-Cre system, since two mouse lines are needed for functional complementation of NCre and CCre, and could result in underestimation of complementation. Indeed, when we directly compared the pattern of recombination in split-Cre (PLP-NCre x PLP-CCre) and full-length Cre (PLP-Cre) transgenic mice, less recombined cells were detected in split-Cre mice (Fig. S3, S4). Similarly, expression of Cre fragments after viral infection is strongly determined by viral serotype and infection rate as well as the activity of chosen promoter elements.
3. Efficiency of split-Cre complementation. The efficiency of split-Cre complementation *in vitro* was lower than that of full-length Cre (Fig. S1); similar as shown for different Cre-fragments *in vitro* [15], [30], [31]. In instances of very short coincident activity of the two promoters, this might cause decreased rates of recombination. Therefore, split-Cre might underestimate the amount of cells with synced promoter activities. But, cells that show DNA-recombination co-expressed NCre and CCre, since in mice expressing only either NCre or CCre no recombination could be observed.
4. Specificity of reporter gene expression. The analysis of Cre mediated recombination depends on the reliability of labelling in the reporter mouse strain used. For example, it has been shown for another ROSA26-based reporter line (ROSA26-loxP-STOP-LoxP-LacZ) [32] that this labels differentiated astrocytes poorly [2], [4]. Therefore, we cannot exclude a biased (i.e. reduced) reporter gene expression in our (ROSA26-loxP-STOP-LoxP-EYFP) ROSY reporter even after successful recombination. This again would result in underestimation of the fraction of astrocytes with recombination. Since abundant recombination in oligodendrocytes and neurons could be detected in the PLP-NCre x PLP-CCre- and the PLP-Cre mice with the same ROSY reporter allele, we can think it highly unlikely that these cell types might have been targeted in GFAP-NCre x PLP-CCre mice, but were not observed due to poor expression of the ROSY reporter allele.

### Split-Cre complementation can be broadly applied

We used two independent strategies to express split-Cre protein fragments in the brains of living mice. First, NCre and CCre were expressed in mice as transgenes driven by the PLP- [33], [34] and the GFAP-promoter [35], [36]. While this approach leads to a stable expression pattern, the generation of transgenic mice is expensive and time consuming. Therefore, we also tested NCre and CCre complementation in adeno-associated virus-mediated gene transfer [37]. When NCre- and CCre were expressed under the control of GAD67 and CCK promoters, functional complementation of split-Cre could be achieved in a subset of interneurons. Therefore, we conclude that split-Cre complementation can be broadly applied and is independent of the expression method as well as the cellular context.

A major strength of split-Cre is its versatility for different experimental approaches due to its unique combination of features, which has not been reported for any other technology so far. Fate mapping experiments based on the overlapping activity of two promoters have been performed previously by using two independent recombinases (Cre and Flp) driven by two promoters combined with a special reporter allele (for review: [38]). This system has been used to analyse the development of several brain regions [39]–[41]. Unfortunately, this strategy is limited to fate mapping rather than the generation of knockout mice by the need for special reporter alleles which is not compatible with the numerous mouse lines harboring LoxP-flanked genes. Xu *et al.* expressed Cre-fragments fused to antiparallel leucine-zipper peptides in the pancreas using the same Pdx1-promoter for both fragments [31]. Jullien *et al*. used constitutive active promoters to drive the expression of rapamycine-sensitive fusion proteins of Cre fragments, generating a new version of inducible Cre. However, this approach is ineffective in the brain since the blood-brain barrier is poorly permeable to rapamycin [16]. Therefore, our study is to the best of our knowledge the first showing DNA recombination induced by complementation of inactive Cre-fragments expressed from two different cell type specific promoters in transgenic mice as well as after adeno-associated viral gene transfer. We prove the widespread applicability to the central nervous system by using two different pairs of DNA regulatory elements which are active in neurons or glia, respectively. Our split-Cre system is ideally suited to be combined with standard LoxP-reporter alleles for lineage fate mapping or selective cell ablation in mice with recombination-induced diphtheria toxin expression [17], [32], [42], [43]. Furthermore, a protein of interest can be expressed from appropriate transgenes using LoxP-flanked STOP-cassettes [6]. Finally, split-Cre is also compatible with the increasing number of mouse lines harboring LoxP-flanked genes for conditional knock-out studies [6].

### A cell population characterized by simultaneous activity of the GFAP- and the PLP-promoter

To evaluate the potency of split-Cre, we analyzed the progeny of GFAP+/PLP+ cells during brain development using the GFAP-NCre x PLP-CCre-mice. These results were compared to the descendants of GFAP-positive as well as PLP-positive cells during brain development, which have been analyzed in detail before [2], [3]. Using PLP-Cre as well as GFAP-Cre mice two phases of embryonic cell differentiation were identified. During early development (E11–E14) embryonic radial glia are neurogenic, while in a later phase after E16 radial glia are gliogenic [2]–[4]. Since the PLP- as well as the GFAP-promoter is already active in the early phase, PLP-Cre and GFAP-Cre mice activate reporter genes in both neurons and glia [2]–[4]. Driving split-Cre expression by either the PLP- or the GFAP- promoter reproduced these findings. However, in GFAP-NCre x PLP-CCre mice split-Cre complementation resulted in recombination only in glia (astrocytes and NG2 glia) and not in neurons. This suggests that either two types of embryonic neural progenitors exist or that the PLP+/GFAP+ cells are generated at a late stage of development, when the neuronal lineage is already separated from the glial lineage, e.g. during the differentiation of PLP+ glia progenitors [3] to astrocytes. The latter is supported by the finding that cells expressing the reporter EYFP appear around E18. In addition, the recombination observed in astrocytes (also in cerebellar Bergmann glia and retinal Müller cells) hints towards a role of oligodendroglial genes (PLP) in early astroglia differentiation, supporting the idea of PLP+ glia progenitors, which give rise to astrocytes [3].

In addition to astrocytes, we find recombination in NG2+ cells of GFAP-NCre x PLP-CCre mice. NG2+ cells have been referred to as oligodendrocyte precursor cells for a long time, but very recent evidence suggests that NG2+ cells (besides numerous other functions [44]) generate oligodendrocytes as well as gray matter astrocytes *in vivo*, the latter most likely via a transition state which is NG2+/GFAP+ [45]. Furthermore, in PLP-EGFP transgenic mice, two populations of NG2+ cells have been described, one expressing the PLP-EGFP transgene, the other being negative for EGFP [46]. Therefore, it is tempting to speculate that the astrocytes, which we found to be labeled in GFAP-NCre x PLP-CCre-mice might be descendants of a NG2+/PLP+ cell population [46], which activates transcription from the GFAP-promoter during differentiation to the astrocytic lineage. While this hypothesis remains to be analyzed in further experiments, split-Cre offers a direct tool to test it by expressing one Cre-fragment under the control of the NG2-promoter [45] and combining these mice with mice expressing the other Cre-fragment under the PLP- as well as the GFAP-promoter, thereby highlighting the powerful potential of split-Cre for the analysis of cell lineages during development.

### Concluding remarks

We present here a powerful tool which allows selective genetic targeting of cell populations defined by the simultaneous activity of two promoters in the mouse, thereby adding a second dimension of cell-type specific control to Cre-based conditional mouse genetics. In addition, split-Cre can be used as a genetic coincidence detector, which converts a temporal co-activity of two promoters in a permanent readout. The split-Cre systems allows for a more precise ‘genetic access’ [47] of (sub-) populations of cells, which will offer advanced analysis of gene and cell function in the mouse brain, as well as in other organs.

## Materials and Methods

### Ethics statement

Mouse breeding in the animal facilities of the Max-Planck Institute of Experimental Medicine and animal experiments including viral infections were performed according to European and German guidelines for the welfare of experimental animals.

### Cloning of split-Cre fusion proteins

The N- (aa 19–59) and C-terminal (aa 60–343) parts of iCre recombinase [48] were PCR amplified, fused to the GCN4 coiled-coil domain [14], a flexible linker (ASPSNPGASNGS; [15]) and a nuclear localisation sequence and cloned into pCMV-Tag-2B or pCMV-Tag-3B (Stratagene, La Jolla, CA, USA), respectively (Fig. 1B). These fusion proteins are referred to as NCre and CCre, respectively. See Supporting Information for details.

### Generation of transgenic mice

Transgenic mice were generated using standard procedures. The expression cassettes encoding NCre and CCre were cloned into well characterised transgene vectors [49]. To drive the cell-specific expression we used regulatory elements from the following genes: *gfap* (homo sapiens, astrocytes, [35]), *plp1* (mus musculus, oligodendrocytes, [33]). Linearized transgenes were injected into the pronucleus of fertilized oocytes obtained from FVB/N-mice. Transgenic animals were crossed to ROSA26-LoxP-STOP-LoxP-EYFP-reporter mice (ROSY; [17]) and recombination was analysed in triple-transgenic animals containing the NCre-, CCre- as well as the reporter allele. See Supporting Information Text S1 for details.

### Generation of adeno-associated viruses

NCre and CCre were separately constructed into recombinant AAV (rAAV) vector plasmid (pAAV-6P-SEWB; [50]), and driven by regulatory elements of the following genes: cholecystokinin, *cck* (mus musculus, 3.0 kb, interneurons), glutamic acid decarboxylase 1, *gad1* (mus musculus, 3.0 kb, interneurons, GAD67) and *gfap* (homo sapiens, 2.2 kb, astrocytes). A cross-packaging of AAV serotype 1 and serotype 2 were performed by triple-transfection of either NCre and CCre type of rAAV vector plasmid and both p-DP1 and p-DP2 plasmids that express the AAV2 rep and cap genes as well as the adenovirus E4, VA, E2a helper regions into HEK 293 cells [37], [51]. Virus was harvested by three times freezing and thawing as described before [37].

### Injection of adeno-associated viruses

Mice were anesthetized using either ketamine (87 mg/kg body weight) and xylazine (13 mg/kg body weight) or avertin (275 mg/kg body weight). Mice were placed into a stereotactic device and 500 nl of a NCre and CCre virus containing solution were injected into the cortex or hippocampus. After surgical dressing, mice were allowed to recover and reporter gene expression was analysed 14 to 21 days later.

### Immunohistochemistry

Immunohistochemistry on vibratome slices of paraformaldehyde-fixed tissue was performed using standard procedures as described [29], [49] and imaged using confocal laser scanning microscopy. In all figures, images of immunostainings using anti-GFP-antibodies (showing expression of the reporter gene EYFP) are shown in green irrespectively of the fluorophor used for detection of the antigen. See Supporting Information Text S1 for antibodies and details.

## Supporting Information

Figure S1 Functional complementation of NCre and CCre in vitro. A: COS1 cells were transfected either with control plasmids (mock), the reporter plasmid CMV-LoxP-STOP-LoxP-EGFP (reporter) or the reporter in combination with full-length Cre (full Cre), NCre or CCre alone or NCre+CCre. Recombination events were monitored using flow cytometry. B: CHO cells were transfected either with control plasmids (mock), the reporter plasmid CMV-LoxP-STOP-LoxP-Luciferase (reporter) or the reporter in combination with full-length Cre (full Cre), NCre or CCre alone or NCre+CCre. Recombination events were monitored using luciferase activity. C: PC12 20.4 cells, which contain a stably integrated CMV-LoxP-STOP-LoxP-EGFP-reporter cassette were transfected either with control plasmids (mock), full-length Cre (full Cre), NCre or CCre alone or NCre+CCre. Recombination events were monitored using flow cytometry scoring for EGFP expression. The figure shows representative experiments (mean±SD; n = 3). All experiments were replicated at least 3 times showing similar results.
(0.11 MB TIF)

Figure S2 Transgene constructs and adeno-associated viral constructs. A: Transgene constructs used for expression of split-Cre in vivo (1: PLP-NCre; 2: PLP-CCre; 3: GFAP-NCre; 4: GFAP-CCre). Due to the very different size of the constructs, the schemes are not scaled. PLP-constructs were linearized using ApaI and SacII, GFAP-constructs using DraIII and ApaLI prior to mouse oocyte injection. B: Constructs for expression of split-Cre using adeno-associated viruses. NCre and CCre were placed under the control of the GFAP-, the CCK- as well as the GAD67-promoter in adeno-associated viral backbone.
(0.26 MB TIF)

Figure S3 Functional Cre complementation of oligodendrocytes but also of neurons and astrocytes in PLP-NCre x PLP-CCre mice. Fixed brain slices of PLP-NCre (line PCND) x PLP-CCre (line PCCR) - mice were analysed for recombination using the ROSY-reporter allele (A, E, H, K, O, R) and counterstained using antibodies against CC1 (B, L), NeuN (F, P) and GFAP (I, S) as markers for oligodendrocytes, neurons and astrocytes, respectively. To compare the pattern of recombination, slice of PLP-Cre x ROSY-mice stained for the reporter EYFP are shown in D and N. Shown are images from the cortex (A–J) and the CA1-region of the hippocampus (K–T). Arrows highlight cells, which show recombination and are positive for the respective marker protein. The scale bar in T corresponds to 50 µm and applies to all panels.
(6.81 MB TIF)

Figure S4 Stable pattern of recombination in PLP-promoter driven transgenic animals between different mouse lines. Different lines of PLP-NCre mice (PCNA, PCND) were crossed to different lines of PLP-CCre mice (PCCK, PCCR) and recombination was analysed using the ROSY reporter allele by EYFP-immunostaining in different brain regions. left column: line PCNA x PCCK. 2nd column: line PCND x PCCK. 3rd column: line PCNA x PCCR. The fourth possible combination (PCCD x PCCR) is already shown in Fig. 1 and Supporting Fig. 3. For comparison, recombination using PLP-Cre-mice is shown in the right column. The pattern of recombination in the cortex (ctx), hippocampus (hc), corpus callosum (cc), cerebellum (cb), cerebellar white matter (cb WM) as well as in the striatum (str) is similar in all four combinations, showing recombination in oligodendrocytes, astrocytes and neurons. However, there are differences in the amount of reporter-labeled cells in the different combinations. The scale bar in X corresponds to 50 µm and applies to all panels.
(4.61 MB TIF)

Figure S5 Widespread Cre complementation in astrocytes, neurons and oligodendrocytes of GFAP-NCre x GFAP-CCre mice. Fixed brain slices of GFAP-NCre (line GCNV) x GFAP-CCre (line GCCF) - mice were analysed for recombination using the ROSY-reporter allele (A, D, G, J, M, P) and counterstained using antibodies against NeuN (B), S100β (E, H, K) and CC1 (N, Q) as markers for neurons, astrocytes and oligodendrocytes, respectively. Shown are images from the cortex (A–F), cerebellum (G–I), hippocampus (J–L), thalamus (M–O) and midbrain (P–R). Arrows highlight cells, which show recombination and are positive for the respective marker protein, while arrowheads mark reporter-labeled cells negative for the marker protein counterstained. The scale bar in R corresponds to 50 µm and applies to all panels.
(5.55 MB TIF)

Figure S6 In GFAP-NCre x PLP-CCre-mice astrocytes are the main population of cells with Cre complementation. Transgenic mouse lines expressing NCre under the GFAP-Promoter (line GCNT) and CCre under the control of the PLP-Promoter (line PCCR) were crossed to ROSY-reporter mice and analysed for recombination (A, D, G, J, M, P). Slices were counterstained using the astroglial marker S100β (B, K), GFAP (E, H), as well as the oligodendroglial marker CC1 (N, Q). Overlays are shown in C, F, I, L, O and R. The preponderant majority of cells with recombination in these mice stain also for the astroglial markers (examples are marked by arrows in A–L). Arrow heads highlight reporter-labeled cells negative for the respective marker protein. No co-localization of EYFP-expression and the oligodendroglial marker CC1 could be found in the cerebellar white matter (M–O) or the corpus callosum (P–R). The scale bar in R corresponds to 50 µm and applies to all panels. str: striatum; hc: hippocampus; cb WM: cerebellar white matter; cc: corpus callosum.
(5.61 MB TIF)

Figure S7 Functional complementation of split-Cre in vivo after viral infection. Adeno-associated viral vectors (AAV) containing NCre or CCre under the control of different promoters were injected into brains of ROSY-reporter mice and recombination was analysed by EYFP-expression. A–F: Co-injection of AAV containing GFAP-NCre and GFAP-CCre leads to abundant recombination in astrocytes along the injection canal and the targeted area. The dotted line in A–C gives the location of the pia mater, the star depicts the site of injection. The box in C indicates the area of the higher magnification images shown in D–F which illustrate that cells with recombination (D) are positive for the astroglial marker GFAP (E; examples highlighted by arrows). G–R: When using the GAD67-promoter to drive NCre as well as CCre expression reporter-labeled interneurons were detected (G, J), which expressed the classical interneuron marker parvalbumin (H, K, arrows). In addition, counterstaining using the neuronal marker NeuN (N, Q) shows that NeuN-positive cells are recombined as analysed by expression of the reporter gene EYFP (M, P) in the dentate gyrus (arrows). Arrowheads mark reporter-labeled cells which are negative for the respective marker protein. The scale bar in R corresponds to 200 µm (panel A–C) or 50 µm (panel D–R). ctx: cortex; cc: corpus callosum; hc: hippocampus; DG: dentate gyrus.
(5.86 MB TIF)

Text S1 Split-Cre in vivo: Supporting Method Details and Tables
(0.07 MB DOC)
