# Supplementary figures and images for: Split-Cre Complementation Indicates Coincident Activity of Different Genes In Vivo
Source: PLoS One. 2009 Jan 27;4(1):e4286. doi: 10.1371/journal.pone.0004286 (PMC2628726; doi:10.1371/journal.pone.0004286)

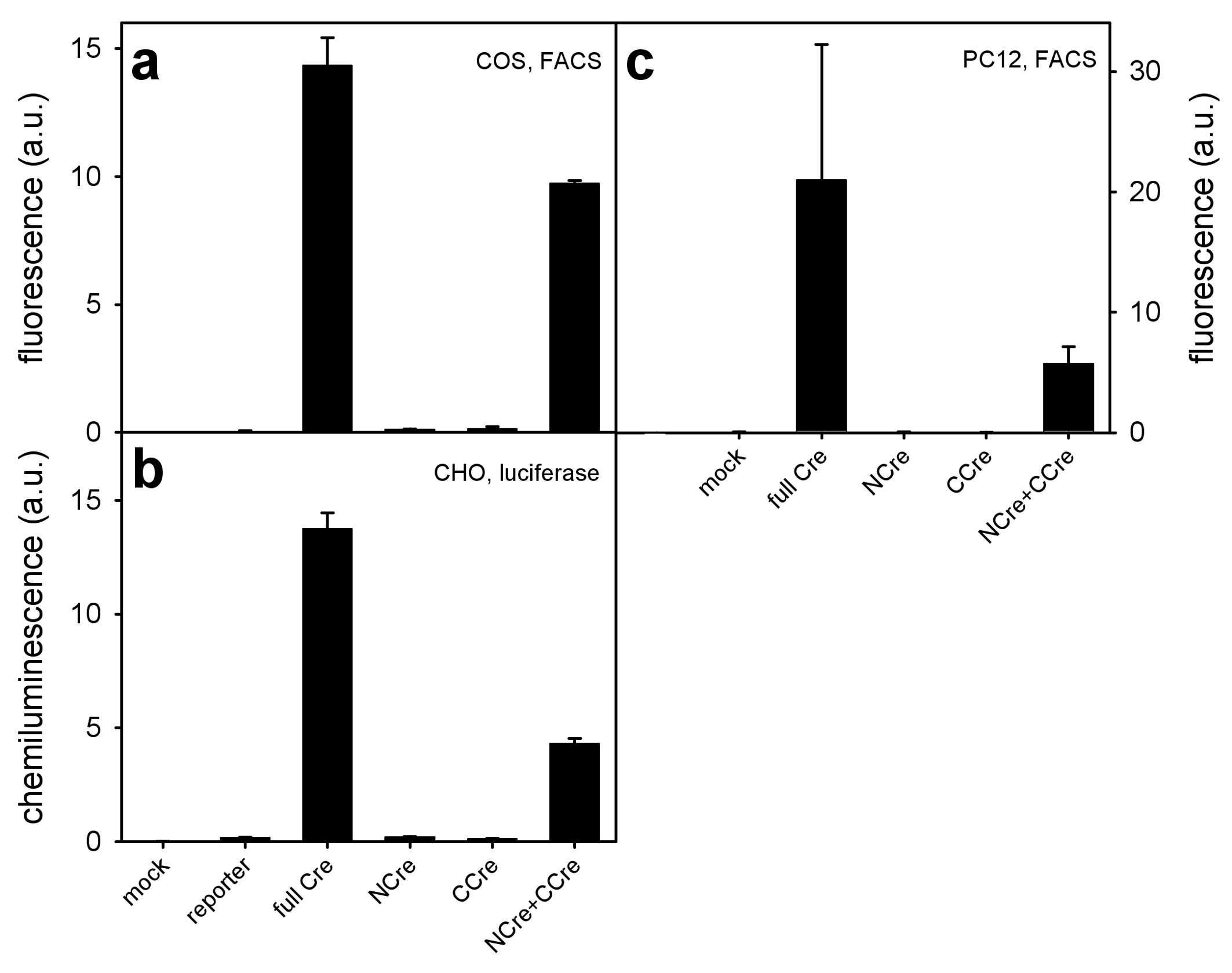

Supplement: Figure S1 — Functional complementation of NCre and CCre in vitro. A: COS1 cells were transfected either with control plasmids (mock), the reporter plasmid CMV-LoxP-STOP-LoxP-EGFP (reporter) or the reporter in combination with full-length Cre (full Cre), NCre or CCre alone or NCre+CCre. Recombination events were monitored using flow cytometry. B: CHO cells were transfected either with control plasmids (mock), the reporter plasmid CMV-LoxP-STOP-LoxP-Luciferase (reporter) or the reporter in combination with full-length Cre (full Cre), NCre or CCre alone or NCre+CCre. Recombination events were monitored using luciferase activity. C: PC12 20.4 cells, which contain a stably integrated CMV-LoxP-STOP-LoxP-EGFP-reporter cassette were transfected either with control plasmids (mock), full-length Cre (full Cre), NCre or CCre alone or NCre+CCre. Recombination events were monitored using flow cytometry scoring for EGFP expression. The figure shows representative experiments (mean±SD; n = 3). All experiments were replicated at least 3 times showing similar results. (0.11 MB TIF) [file pone.0004286.s001.tif]

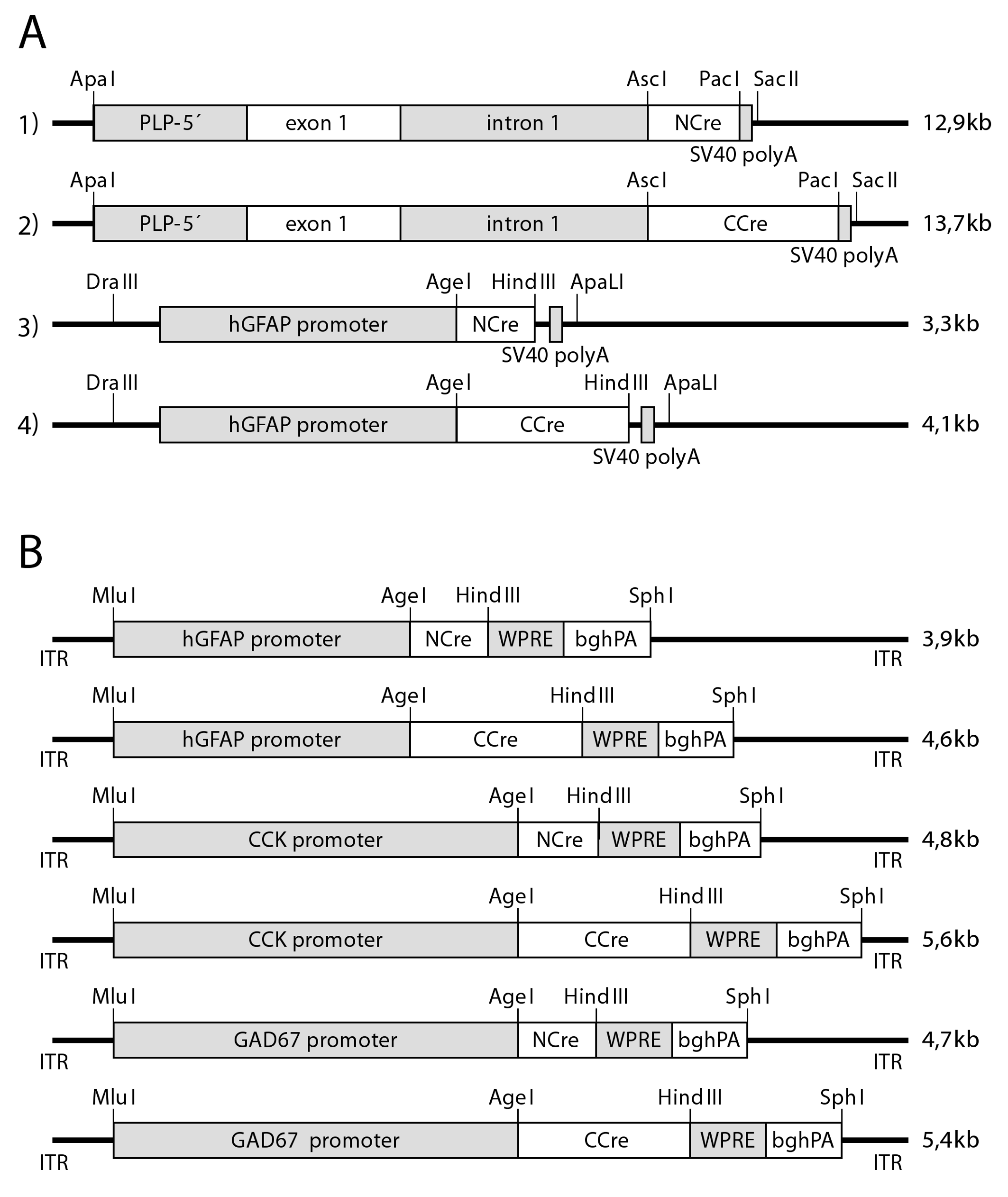

Supplement: Figure S2 — Transgene constructs and adeno-associated viral constructs. A: Transgene constructs used for expression of split-Cre in vivo (1: PLP-NCre; 2: PLP-CCre; 3: GFAP-NCre; 4: GFAP-CCre). Due to the very different size of the constructs, the schemes are not scaled. PLP-constructs were linearized using ApaI and SacII, GFAP-constructs using DraIII and ApaLI prior to mouse oocyte injection. B: Constructs for expression of split-Cre using adeno-associated viruses. NCre and CCre were placed under the control of the GFAP-, the CCK- as well as the GAD67-promoter in adeno-associated viral backbone. (0.26 MB TIF) [file pone.0004286.s002.tif]

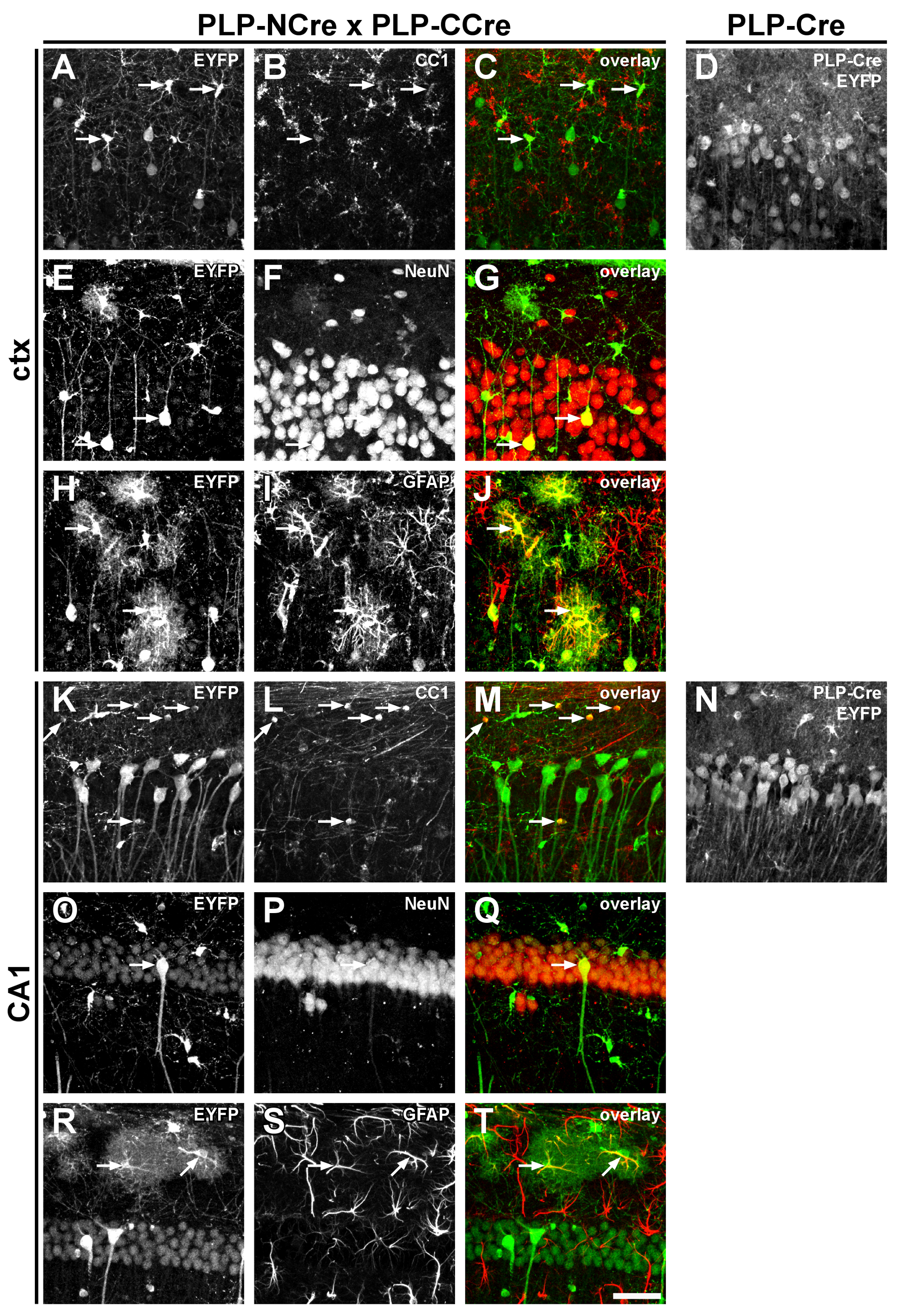

Supplement: Figure S3 — Functional Cre complementation of oligodendrocytes but also of neurons and astrocytes in PLP-NCre x PLP-CCre mice. Fixed brain slices of PLP-NCre (line PCND) x PLP-CCre (line PCCR) - mice were analysed for recombination using the ROSY-reporter allele (A, E, H, K, O, R) and counterstained using antibodies against CC1 (B, L), NeuN (F, P) and GFAP (I, S) as markers for oligodendrocytes, neurons and astrocytes, respectively. To compare the pattern of recombination, slice of PLP-Cre x ROSY-mice stained for the reporter EYFP are shown in D and N. Shown are images from the cortex (A–J) and the CA1-region of the hippocampus (K–T). Arrows highlight cells, which show recombination and are positive for the respective marker protein. The scale bar in T corresponds to 50 µm and applies to all panels. (6.81 MB TIF) [file pone.0004286.s003.tif]

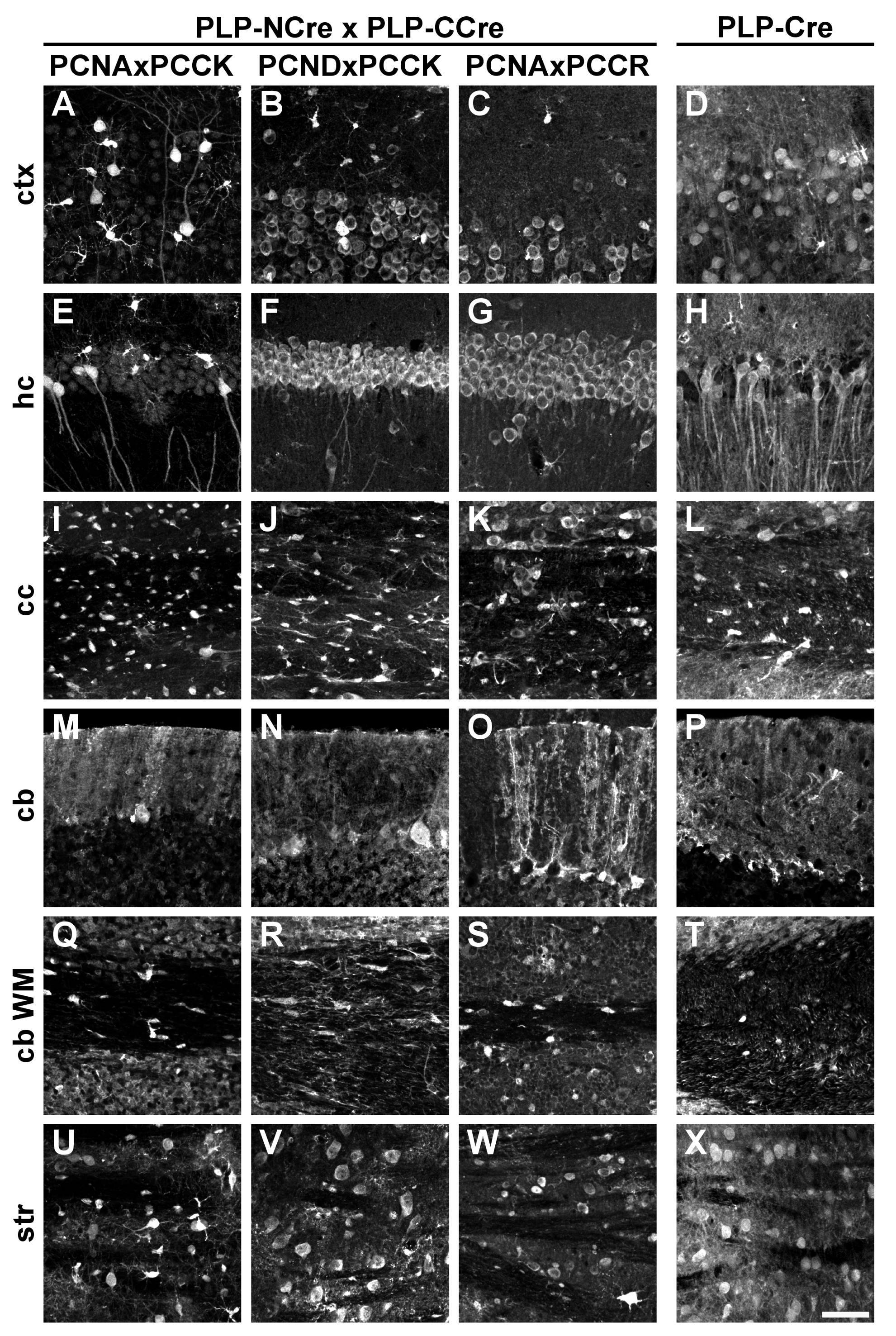

Supplement: Figure S4 — Stable pattern of recombination in PLP-promoter driven transgenic animals between different mouse lines. Different lines of PLP-NCre mice (PCNA, PCND) were crossed to different lines of PLP-CCre mice (PCCK, PCCR) and recombination was analysed using the ROSY reporter allele by EYFP-immunostaining in different brain regions. left column: line PCNA x PCCK. 2nd column: line PCND x PCCK. 3rd column: line PCNA x PCCR. The fourth possible combination (PCCD x PCCR) is already shown in Fig. 1 and Supporting Fig. 3. For comparison, recombination using PLP-Cre-mice is shown in the right column. The pattern of recombination in the cortex (ctx), hippocampus (hc), corpus callosum (cc), cerebellum (cb), cerebellar white matter (cb WM) as well as in the striatum (str) is similar in all four combinations, showing recombination in oligodendrocytes, astrocytes and neurons. However, there are differences in the amount of reporter-labeled cells in the different combinations. The scale bar in X corresponds to 50 µm and applies to all panels. (4.61 MB TIF) [file pone.0004286.s004.tif]

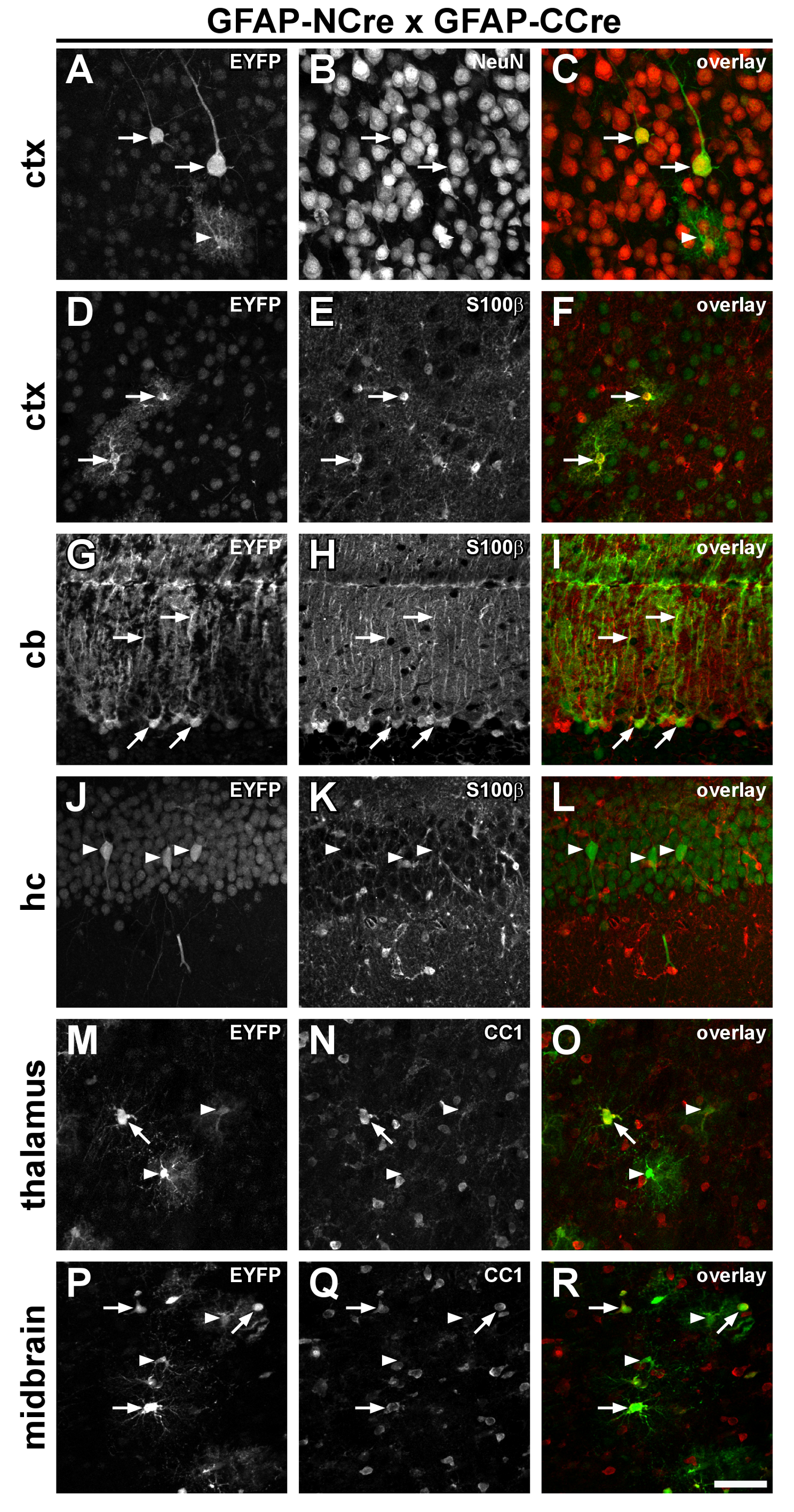

Supplement: Figure S5 — Widespread Cre complementation in astrocytes, neurons and oligodendrocytes of GFAP-NCre x GFAP-CCre mice. Fixed brain slices of GFAP-NCre (line GCNV) x GFAP-CCre (line GCCF) - mice were analysed for recombination using the ROSY-reporter allele (A, D, G, J, M, P) and counterstained using antibodies against NeuN (B), S100β (E, H, K) and CC1 (N, Q) as markers for neurons, astrocytes and oligodendrocytes, respectively. Shown are images from the cortex (A–F), cerebellum (G–I), hippocampus (J–L), thalamus (M–O) and midbrain (P–R). Arrows highlight cells, which show recombination and are positive for the respective marker protein, while arrowheads mark reporter-labeled cells negative for the marker protein counterstained. The scale bar in R corresponds to 50 µm and applies to all panels. (5.55 MB TIF) [file pone.0004286.s005.tif]

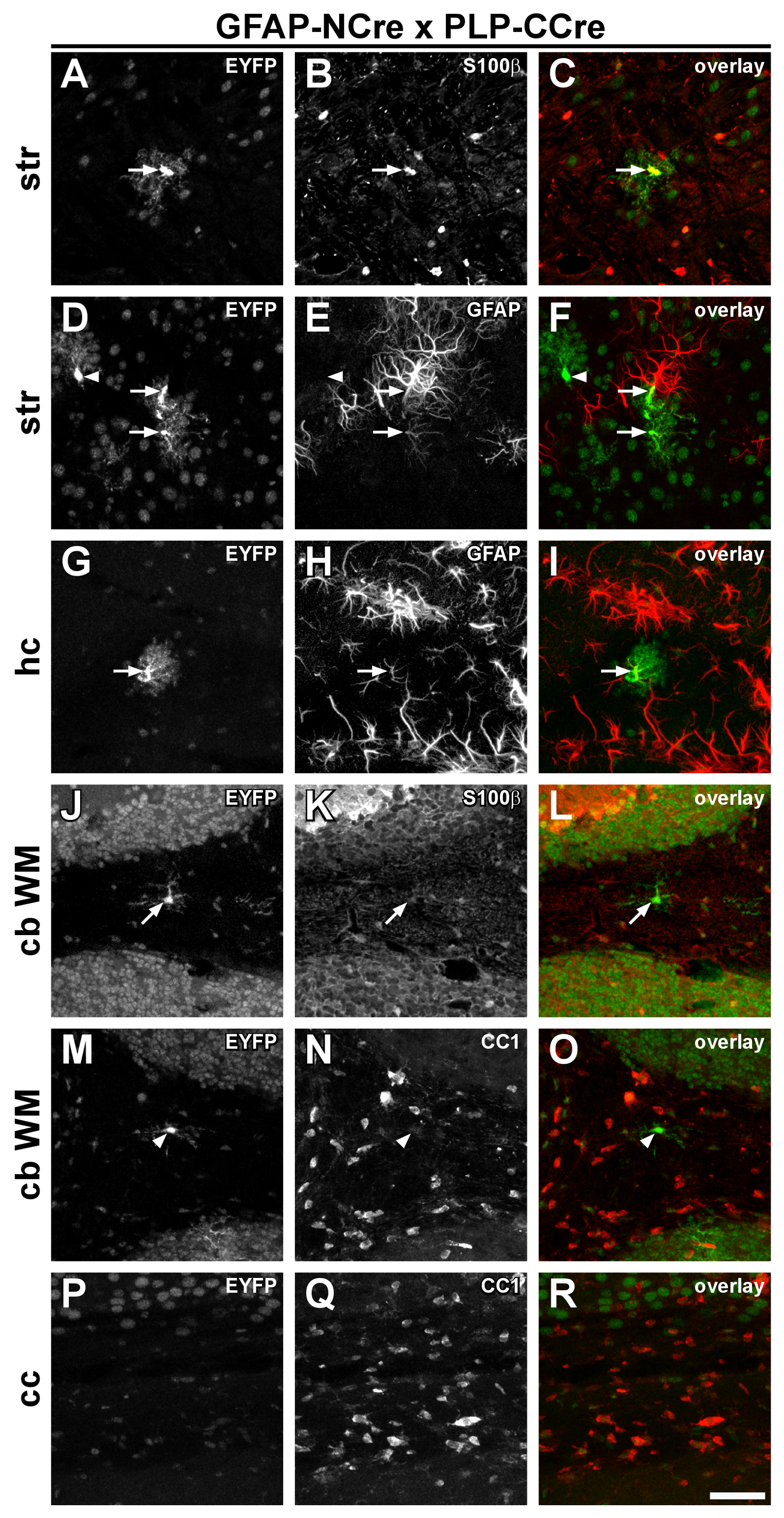

Supplement: Figure S6 — In GFAP-NCre x PLP-CCre-mice astrocytes are the main population of cells with Cre complementation. Transgenic mouse lines expressing NCre under the GFAP-Promoter (line GCNT) and CCre under the control of the PLP-Promoter (line PCCR) were crossed to ROSY-reporter mice and analysed for recombination (A, D, G, J, M, P). Slices were counterstained using the astroglial marker S100β (B, K), GFAP (E, H), as well as the oligodendroglial marker CC1 (N, Q). Overlays are shown in C, F, I, L, O and R. The preponderant majority of cells with recombination in these mice stain also for the astroglial markers (examples are marked by arrows in A–L). Arrow heads highlight reporter-labeled cells negative for the respective marker protein. No co-localization of EYFP-expression and the oligodendroglial marker CC1 could be found in the cerebellar white matter (M–O) or the corpus callosum (P–R). The scale bar in R corresponds to 50 µm and applies to all panels. str: striatum; hc: hippocampus; cb WM: cerebellar white matter; cc: corpus callosum. (5.61 MB TIF) [file pone.0004286.s006.tif]

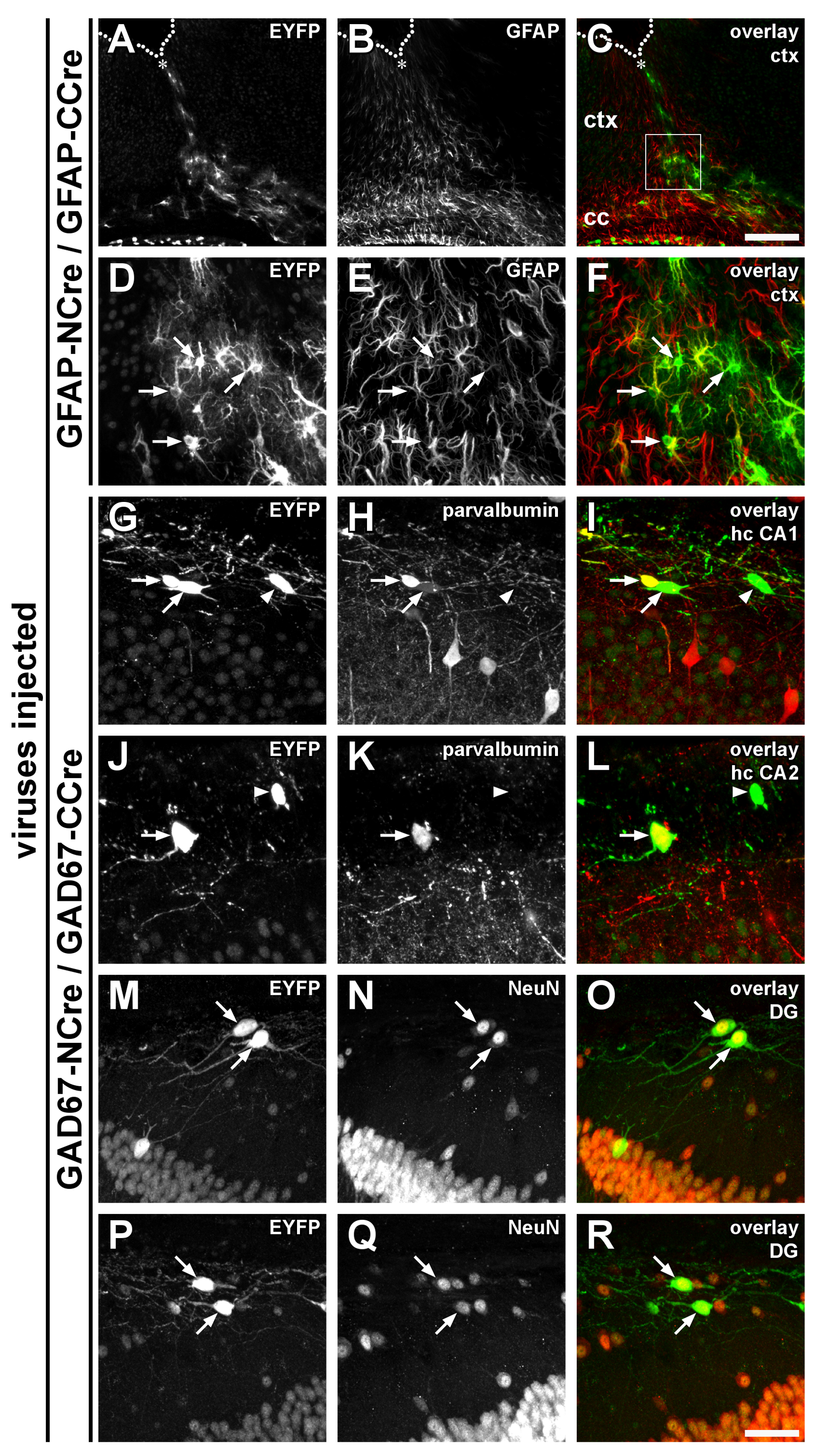

Supplement: Figure S7 — Functional complementation of split-Cre in vivo after viral infection. Adeno-associated viral vectors (AAV) containing NCre or CCre under the control of different promoters were injected into brains of ROSY-reporter mice and recombination was analysed by EYFP-expression. A–F: Co-injection of AAV containing GFAP-NCre and GFAP-CCre leads to abundant recombination in astrocytes along the injection canal and the targeted area. The dotted line in A–C gives the location of the pia mater, the star depicts the site of injection. The box in C indicates the area of the higher magnification images shown in D–F which illustrate that cells with recombination (D) are positive for the astroglial marker GFAP (E; examples highlighted by arrows). G–R: When using the GAD67-promoter to drive NCre as well as CCre expression reporter-labeled interneurons were detected (G, J), which expressed the classical interneuron marker parvalbumin (H, K, arrows). In addition, counterstaining using the neuronal marker NeuN (N, Q) shows that NeuN-positive cells are recombined as analysed by expression of the reporter gene EYFP (M, P) in the dentate gyrus (arrows). Arrowheads mark reporter-labeled cells which are negative for the respective marker protein. The scale bar in R corresponds to 200 µm (panel A–C) or 50 µm (panel D–R). ctx: cortex; cc: corpus callosum; hc: hippocampus; DG: dentate gyrus. (5.86 MB TIF) [file pone.0004286.s007.tif]
