# Supplementary material for: Split-Cre Complementation Indicates Coincident Activity of Different Genes In Vivo
Source: PLoS One. 2009 Jan 27;4(1):e4286. doi: 10.1371/journal.pone.0004286 (PMC2628726; doi:10.1371/journal.pone.0004286)
Supplement: Text S1 — Split-Cre in vivo: Supporting Method Details and Tables (0.07 MB DOC) [file pone.0004286.s008.doc]

**Supporting Information**

**Methods**

**Cloning of split-Cre fusion proteins**

The N- (amino acid (aa) 19–59) and C-terminal (aa 60–343) fragments of iCre recombinase [1] were PCR amplified using primers adding a nuclear localization sequence (NLS; amino acid sequence PKKKRKV; [2]) and BamHI and PstI restriction sites and cloned into pCMV-Tag-2B or pCMV-Tag-3B (Stratagene, La Jolla, CA, USA), respectively, using the BamHI and PstI restriction sites. The GCN4 coiled-coil domain [3] was PCR amplified using primers adding a flexible linker (ASPSNPGASNGS) [4] and EcoRI and HindIII restriction sites and cloned into the same vectors using these restriction sites. The expression constructs consist of the CMV promoter driving the expression of Flag-GCN4-linker-NLS-Cre(aa19-59) or Myc-GCN4-linker-NLS-Cre(aa60-343) (**Fig. 1B**). These fusion proteins are referred to as NCre and CCre, respectively.

**Cell culture and transfection**

COS1 cells were cultured in Dulbecco’s modified Eagle Medium (Gibco) supplemented with 10% fetal bovine serum, 1% penicillin/streptomycin and 1% L-glutamine. CHO cells were cultured in F12 HAM Medium supplemented with 10 % fetal bovine serum, 1% penicillin/streptomycin and 1% L-glutamine. PC12 20.4 cells, stably transfected with a CMV-LoxP-STOP-LoxP-EGFP reporter cassette [3] were cultured in Dulbecco’s modified Eagle Medium (Gibco) supplemented with 10% fetal bovine serum, 10% horse serum, 1% penicillin/streptomycin and 1% L-glutamine. For luciferase-based recombination assay cells were seeded in 96 well plates, for analysis using flow cytometry 24 well plates were used. Cells were transiently transfected with Lipofectamin^TM^ 2000 (Invitrogen) at approximately 75% density. DNA amounts of plasmids were adjusted for transfection to an equal level with pcDNA3.

**Flow cytometry**

COS1- or PC12 20.4 cells were seeded in 24 well plates and transfected with the appropriate combination of plasmids (COS1, PC12 20.4) including a CMV-LoxP-STOP-LoxP-EGFP reporter plasmid (COS1). PC12 20.4 cells contain a stably integrated CMV-LoxP-STOP-LoxP-EGFP reporter cassette [3]. After 48 hours cells were trypsinized on ice, filtered and analysed as a single cell suspension in a flow cytometer (BD FACS Aria; BD Biosciences, Heidelberg, Germany). Results were analysed and visualized using the Software BD FACSDiva™ (BD Biosciences, Heidelberg, Germany). All experiments were repeated at least three times with a minimum of three replicates for each data point.

**Luciferase assays**

Luciferase assays were performed in 96 well plates. Cells were transiently co-transfected with pairs of plasmids including a CMV-LoxP-STOP-LoxP-Firefly-luciferase reporter plasmid and non-regulated Renilla luciferase plasmids for standardisation. After 24 hours of incubation media were removed, 30 µl of passive lysis buffer (Promega, Mannheim, Germany) was added to the wells and cells were lysed for 30 min. The plate was transferred into the luminometer (Microlumatplus LB96Vreader, Berthold Technologies, Bad Wildbad, Germany). 50 µl of Firefly-luciferase substrate solution was automatically added to the wells and luminescence was measured for 6 seconds. 100 µl of solution terminating the Firefly-luciferase-reaction and containing the substrate for Renilla-luciferase was added to each well and luminescence was measured for further 2.5 seconds. Data were analysed using WinGlow (Berthold Technologies) and Excel (Microsoft). Firefly luminescence was normalized to Renilla luminescence to correct for potential heterogeneity of transfection efficiency. All experiments were repeated at least three times with a minimum of three replicates for each data point.

**Generation of transgenic mice**

For generation of transgenic mice, the expression cassettes encoding NCre as well as CCre were cloned into well characterised transgene vectors [5]. To drive the astrocyte specific expression we used the human GFAP promoter [6]. NCre and CCre were PCR-amplified adding an AgeI restriction site as well as a Kozak consensus sequence to the 5’-end and cloned into pGFAP-ECFP-c1 [5] using the AgeI and HindIII restriction sites, thereby removing ECFP from the parent vector. The constructs were controlled by sequencing and linearised with the restriction enzymes DraIII and ApaLI for injection into murine oocytes.

Oligodendroglial expression was achieved by using the murine PLP minigene. The internal ApaI-site within the coding sequence of CCre was removed by site-directed mutagenesis introducing a silent mutation and NCre and CCre were PCR-amplified adding AscI and PacI restriction sites as well as a Kozak consensus sequence. PCR-products were cloned into a vector containing a PLP-minigene (a kind gift of B. Fuss, [7]) using these restriction site. The constructs were controlled by sequencing and linearised with the restriction enzymes ApaI and SacII for injection into murine oocytes.

Transgenic mice were generated by injection of their respective linear transgene-containing construct into the pronucleus of fertilized oocytes obtained from FVB/N mice. Putative transgenic founders were identified by PCR genotyping of tail-tip DNA and subsequently crossbred to wild-type and Cre reporter mice. Positively genotyped F1 litters of transgenic founders were screened for functional expression by Western blotting as well as crossing to appropriate complementary split-Cre mice. All transgene constructs gave rise to functional expression in adult mice. Transgenic animals were crossed to ROSA26-LoxP-STOP-LoxP-EYFP-reporter mice (ROSY; [8]) and recombination was analysed in triple-transgenic animals containing the NCre-, CCre- as well as the reporter allele. As controls, double transgenic animals expressing only one Cre-fragment and the reporter were analysed. In none of these mice recombination could be detected (data not shown).

**Genotyping of split-Cre transgenic mice**

Genotyping of split-Cre mice was done by PCR using combinations of promoter-specific sense primers in combination with NCre- or CCre-specific antisense primers (Supplementary table 1) using tail-tip DNA as template. 1 µl of DNA was amplified in a total volume of 20 µl containing 5 pmol of each primer and 0.7 U RedTaq-Polymerase (Sigma, Deisenhofen, Germany) using the following protocol: 3 min 94°C, 35 or 40 cycles of 30 sec 94°C, 30 sec 60°C and 1 min 72°C each and a final elongation of 10 min at 72°C. Products were analysed by agarose gel electrophoresis.

**Tissue fixation and immunohistochemistry**

Adult mice were deeply anesthetized and perfused transcardially with Hank´s Balanced Salt Solution (HBSS, Gibco) followed by perfusion with 4% paraformaldehyde (PFA) in phosphate buffer for 15 min. The brain was removed and incubated in PFA over night at 4°C. For the preparation of retina, eyes were removed from perfusion-fixed mice and post-fixed in PFA over night. Retinas were carefully prepared and embedded in 3% agarose in phosphate-buffered saline (PBS) prior to slicing. For the preparation of embryos, mice were sacrificed, embryos were prepared and fixed by immersion in PFA over night. Immuno­histochemical labelling was performed on 30 – 60µm free-floating sagittal or horizontal vibratome sections (Leica VT 1000S, Leica Instruments, Nussloch, Germany) at room temperature. Cells were permeabilized with 0.4% Triton-X100 in PBS for 30 min and then blocked for 30 min in 4 % horse serum (HS) with 0.2% Triton-X100 in PBS. The slices were incubated over one to three nights at 4°C in 1% HS and 0.05% Triton-X100 in PBS with the first antibodies (Supplementary Table 2). Slices were washed twice in PBS for 10 min and incubated with Cy2-, Cy3 or Cy5-conjugated secondary antibodies (Supplementary Table 2) for 2h in 1.5% HS in PBS. After washing twice in PBS and once in water, slices were mounted with Immu-Mount (Shandon).

**Confocal laser scanning microscopy**

Sections were investigated by confocal laser-scanning microscopy (Zeiss LSM 510NLO, Axiovert 200M or Axioskop FS2mot) equipped with laser lines at 458/488/514, 543 and 633 nm. Three-dimensional image stacks were recorded with frame sizes of 1024 x 1024 pixels (voxel size between 0.21 µm x 0.21 µm x 1.0 µm and 0.23 µm x 0.23 µm x 2.5 µm) using a C-Apochromat 40x/1.2NA or Plan-Neofluar 40x/1.3NA objective (Zeiss). All confocal images were stored and processed with the Zeiss LSM Software. Images were filtered using a median filter to remove noise, and maximum intensity projections spanning 10 to 50 µm were calculated. Images were then exported into Adobe Photoshop, filtered using the dust and scratches-filter (radius 1 pixel). Contrast and brightness were adjusted to allow proper visualization and overlay images were generated. Final figures were assembled using Adobe InDesign.

**Supporting References**

1. Shimshek DR, Kim J, Hubner MR, Spergel DJ, Buchholz F, Casanova E, Stewart AF, Seeburg PH, Sprengel R (2002) Codon-improved Cre recombinase (iCre) expression in the mouse. Genesis 32: 19-26.

2. Kalderon D, Roberts BL, Richardson WD, Smith AE (1984) A short amino acid sequence able to specify nuclear location. Cell 39: 499-509.

3. Wehr MC, Laage R, Bolz U, Fischer TM, Grunewald S, Scheek S, Bach A, Nave KA, Rossner MJ (2006) Monitoring regulated protein-protein interactions using split TEV. Nat Methods 3: 985-993.

4. Jullien N, Sampieri F, Enjalbert A, Herman JP (2003) Regulation of Cre recombinase by ligand-induced complementation of inactive fragments. Nucleic Acids Res 31: e131.

5. Hirrlinger PG, Scheller A, Braun C, Quintela-Schneider M, Fuss B, Hirrlinger J, Kirchhoff F (2005) Expression of reef coral fluorescent proteins in the central nervous system of transgenic mice. Mol Cell Neurosci 30: 291-303.

6. Brenner M, Kisseberth WC, Su Y, Besnard F, Messing A (1994) GFAP promoter directs astrocyte-specific expression in transgenic mice. J Neurosci 14: 1030-1037.

7. Fuss B, Mallon B, Phan T, Ohlemeyer C, Kirchhoff F, Nishiyama A, Macklin WB (2000) Purification and analysis of in vivo-differentiated oligodendrocytes expressing the green fluorescent protein. Dev Biol 218: 259-274.

8. Srinivas S, Watanabe T, Lin CS, William CM, Tanabe Y, Jessell TM, Costantini F (2001) Cre reporter strains produced by targeted insertion of EYFP and ECFP into the ROSA26 locus. BMC Dev Biol 1: 4.

**Supporting Tables**

**Table S1: Genotyping of split-Cre mice**

A: Primer used

| **Number** | **position** | **direction** | **Sequence (5’🡪3’)** |
| --- | --- | --- | --- |
| 4750 | GFAP-promoter | sense | CAGGTTGGAGAGGAGACGCATCA |
| 3471 | PLP-promoter | sense | ATGCGTACCTGACTTTCTCCTTCT |
| 7983 | N-Cre | antisense | CCATCAGGTTCTTCCTGACTTCATCA |
| 7984 | C-Cre | antisense | CTCCATCAGGGATCTGACTTGGTCAA |

**B: Primer combinations**

| **genotype** | **sense** | **antisense** | **Product size (bp)** |
| --- | --- | --- | --- |
| GFAP-NCre | 4750 | 7983 | 384 |
| GFAP-CCre | 4750 | 7984 | 631 |
| PLP-NCre | 3471 | 7983 | 361 |
| PLP-CCre | 3471 | 7984 | 608 |

**Table S2**: Source and dilution of antibodies

| **Antibody** | **Source** | **Dilution used** |
| --- | --- | --- |
| Mouse anti-APC, clone CC1 | Calbiochem | 1:50 |
| Rabbit anti-calretinin | Chemicon | 1:1000 |
| Mouse anti-CRALBP | Acris | 1:500 |
| Mouse anti-GFAP | Novocastra | 1:200 |
| Rabbit anti-GFP | Abcam | 1:1000 |
| Goat anti-GFP | Rockland | 1:1000 |
| Mouse anti-NeuN | Chemicon | 1:100 |
| Rat anti-NG2 | Dr. J. Trotter | 1:500 |
| Mouse anti-parvalbumin | Sigma | 1:500 |
| Rabbit anti-S100β | Swant | 1:500 |
| Cy2, Cy3 or Cy5 conjugated  secondary antibodies | Dianova | 1:100 (Cy2)  1:1000 (Cy3, Cy5) |
| Alexa488 or Alexa546 conjugated secondary antibodies | Invitrogen | 1:2000 |

**Table S3: Mouse lines used in this study**

| **Transgene** | **Lines** |
| --- | --- |
| PLP-NCre | PCNA PCNC PCND |
| PLP-CCre | PCCK PCCR |
| GFAP-NCre | GCNQ GCNT GCNV GCNW |
| GFAP-CCre | GCCF |
| PLP-Cre | PLCF |
| ROSA26-LoxP-STOP-LoxP-EYFP | ROSY |
